# Late-onset Male Hypogonadism and Fertility Potential in Thalassemia Major Patients: Two Emerging Issues

**DOI:** 10.4084/MJHID.2015.047

**Published:** 2015-07-10

**Authors:** Vincenzo De Sanctis, Ashraf T. Soliman, Heba Elsedfy, Nada A. Soliman, Rania Elalaily

**Affiliations:** 1 Pediatric and Adolescent Outpatient Clinic, Quisisana Hospital, Ferrara, Italy; 2 Department of Pediatrics, Division of Endocrinology, Alexandria University Children’s Hospital, Alexandria; 3 Department of Pediatrics, Ain Shams University, Cairo, Egypt; 4 Ministry of Health, Alexandria, Egypt; 5 Department of Primary Health Care, Abu Nakhla Hospital, Doha, Qatar

## Dear Sir

Today many subjects with beta thalassaemia major (β-thal) successfully survive into adult life, due to the remarkable improvement of medical care and better understanding of pathogenesis, clinical manifestations and prevention of endocrine complications.^1^ However, involvement of the endocrine system still burdens the life of these patients. In fact, several studies have reported that as many as 51% to 66% of patients may have pubertal failure, sexual dysfunction and infertility, due to hypogonadism.^1^,^2^

An emerging endocrine disorder in young adults with β-thal is late-onset male hypogonadism (LOH). LOH is a disorder caused by the inability of the testes to produce the physiologic levels of testosterone and normal number of spermatozoa as a result of a disruption of the hypothalamic- pituitary-gonadal axis . Ten out of our 120 β-thal patients (8.3%) developed this complication and 35% developed hypogonadotropic hypogonadism requiring sex hormone replacement therapy.^3^

The causes of male hypogonadism in the general population are multiple. In β-thal hypogonadism is mainly due to iron deposition in the endocrine glands.

Iron overload may be the result of economic circumstances (high expense of the chelation therapy), late onset of chelation therapy or poor compliance with treatment.

Toxicity starts when the iron load in a particular tissue exceeds the tissue or blood-binding capacity of iron and consequently free non-transferrin iron appears. The ‘free iron’ is a catalyst for the production of oxygen species that peroxidise membrane lipids of cell organelles leading to cell destruction.^2^,^3^

The anterior pituitary gland is particularly sensitive to the free radicals produced by oxidative stresses and exposure to these radicals injures the gland. Magnetic resonance imaging (MRI) shows that even a modest amount of iron deposition within the anterior pituitary can interfere with its function. Excess iron deposition in the anterior pituitary leads to degranulation of the adenohypophysis and decreases hormone storage along with hypo-responsiveness to hypothalamic releasing hormones.^1^ This causes:

1. Abnormalities in the hypothalamic- pituitary growth hormone axis (defective GH secretion in response to stimulation(40%) and GH neuro-secretory dysfunction (up to 20%) and low insulin-like growth factor-1 (IGF-1) synthesis in response to GH).^1^
2. Abnormalities in the hypothalamic- pituitary- gonadal axis (hypogonadotropic hypogonadism).^2^ Patients with TM have lower basal FSH and LH secretion, low LH/FSH response to GnRH (gonadotropin releasing hormone) and low sex steroid secretion from the gonads (testosterone). In addition, there is a variable disturbance of the spontaneous pulsatile pattern of LH and FSH secretion (pulsatile versus apulsatile).Those patients with pulsatile secretion of LH/FSH have higher basal LH /FSH, GnRH stimulated LH and FSH, lower ferritin and less organ damage (liver and heart) with better prognosis for fertility. They display complete or partial reversibility of hypogonadism when treated with pulsatile subcutaneous GnRH infusions every 120 minutes for 3 months. While those with apulsatile secretion or poor pulsatile release of LH/FSH do not respond to GnRH infusion and have poor prognosis for fertility (V De Sanctis and AT Soliman, personal observations)

Combined iron chelation therapy (use of two chelators on the same day), may induce negative iron balance and may reverse hypogonadism and endocrine complications in severe iron overloaded β-thal subjects. Long-term studies have shown that the combined use of deferiprone and deferoxamine (DFO) hastens iron chelation by rapidly reducing liver iron, serum ferritin, and myocardial siderosis. Combined chelation therapy with deferasirox and DFO has also been proved beneficial.^1^

## What we know about fertility potential in thalassaemia? The personal experience

Histologically, a reduced number of cells and moderate siderosis of the parenchymal cells of the anterior pituitary have been found. 2 Testicular biopsies display various degrees of interstitial fibrosis and hyperpigmentation of undifferentiated seminiferous tubules and a decreased number of Leydig cells.

Virtually very little is known about spermatogenesis in (β-thal). A summary of the available findings include the followings:

A normal sperm count and motility in 45% of fully sexually mature β-thal subjects.^3^

- A possible detrimental effect of iron chelation therapy on spermatogenesis. Three out of four patients with serum ferritin levels lower than 500 ng/ml had poor sperm motility.^4^,^5^
- A higher degree of defective chromatin packaging in β-thal subjects with low sperm concentrations. 5
- A low seminal plasma concentration of zinc, citric acid and prostate specific antigen. These data suggest impaired prostatic secretion.^6^
- An increase of seminal lipo-peroxidation.^7^
- An increase of DNA sperm damage and a negative correlation with sperm motility. These findings suggest that iron overload predispose sperm to oxidative injury. 8
- These findings indicate an increase of oxidative stress in the semen of these patients that could contribute to the impairment of sperm motility.^6^,^7^
- Blood transfusion is associated with significant acute enhancement of sperm parameters and increased concentrations of serum T, LH, FSH, and IGF-1. These “acute” effects on spermiogenesis are reached by an unknown mechanism and suggest a number of pathways that need further human and/or animal studies.^9^

In addition, abnormal seminal parameters and low serum folic acid levels have been found in subjects with thalassaemia intermedia.^10^

In our experience, β-thal patients develop LOH in their second and third decades of life. It is possible to induce or restore spermatogenesis with exogenous gonadotrophins in some of them.^11^ Assisted reproductive techniques may supplementary help these patients to overcome previously untreatable causes of male infertility.^1^ International guidelines are required to assist these patients because it is widely accepted that infertility and involuntary childlessness, and the decision to engage with assisted reproduction technology services as a patient, donor or surrogate can entail wide-ranging psychosocial issues.

Despite the fact that endocrine complications are very common in multi-transfused thalassaemia patients^1^,^2^,^12^ a recent survey conducted by the International Network of Clinicians for Endocrinopathies in Thalassemia and Adolescent Medicine (ICET-A) in 2014 in Acitrezza(Catania, Italy)showed that the major difficulties reported by hematologists or pediatricians experienced in thalassaemias or thalassaemia syndromes in following endocrine complications were: 1. Lack of familiarity with medical treatment of endocrine complications, 2.poor interpretation of endocrine tests and 3. lack of collaboration and on-time consultation between thalassaemic centers supervised by hematologists and endocrinologists

The practical objectives of ICET-A (www.endothalassemia.org) are to encourage and guide endocrine follow up of multi-transfused patients in developing countries, to promote and support collaborative research in this field, to encourage and guide endocrine follow up of multi-transfused patients, and to educate and train more endocrinologists and other paediatricians/physicians to prevent and improve management of the growth and endocrine complications in these patients.^13^ This is in agreement with a fundamental requirement of medical ethics, that any progress we make in research into growth disorders and endocrine complications in thalassaemia should be passed on to all those suffering from such disorders.
